# Epigallocatechin Gallate Remodelling of Hfq Amyloid-Like Region Affects *Escherichia coli* Survival

**DOI:** 10.3390/pathogens7040095

**Published:** 2018-12-01

**Authors:** David Partouche, Florian Turbant, Omar El Hamoui, Camille Campidelli, Marianne Bombled, Sylvain Trépout, Frank Wien, Véronique Arluison

**Affiliations:** 1 Laboratoire Léon Brillouin LLB, CEA, CNRS UMR12, Université Paris Saclay, CEA Saclay, 91191 Gif-sur-Yvette, France; davidmeyer.partouche@gmail.com (D.P.); flo.turbant@gmail.com (F.T.); c.campidelli@gmail.com (C.C.); Marianne.BOMBLED@cea.fr (M.B.); 2 Synchrotron SOLEIL, L’Orme des Merisiers, Saint Aubin BP48, 91192, Gif-sur-Yvette, France; elhamoui.omar@gmail.com; 3 Institut Curie, INSERM U1196, and CNRS UMR9187, 91405 Orsay Cedex, France; sylvain.trepout@curie.fr; 4 UFR SDV, Université Paris Diderot, Sorbonne Paris Cité, 75013 Paris, France

**Keywords:** bacterial amyloid, functional amyloid, protein fibrils, epigallocatechin gallate (EGCG), protein fibrillation inhibition, Hfq, bacterial adaptation, antibacterial

## Abstract

Hfq is a pleiotropic regulator that has key roles in the control of genetic expression. The protein noticeably regulates translation efficiency and RNA decay in Gram-negative bacteria, due to the Hfq-mediated interaction between small regulatory noncoding RNA and mRNA. This property is of primary importance for bacterial adaptation and virulence. We have previously shown that the Hfq *E. coli* protein, and more precisely its C-terminal region (CTR), self-assembles into an amyloid-like structure. In the present work, we demonstrate that epigallocatechin gallate (EGCG), a major green tea polyphenol compound, targets the Hfq amyloid region and can be used as a potential antibacterial agent. We analysed the effect of this compound on Hfq amyloid fibril stability and show that EGCG both disrupts Hfq-CTR fibrils and inhibits their formation. We show that, even if EGCG affects other bacterial amyloids, it also specifically targets Hfq-CTR in vivo. Our results provide an alternative approach for the utilisation of EGCG that may be used synergistically with conventional antibiotics to block bacterial adaptation and treat infections.

## 1. Introduction

Antibiotic resistance occurs when a compound has lost its ability to kill bacteria. This problem is particularly critical for public health and many efforts have been done to modify and improve existing antibiotics. Indeed, antibacterial agents use four main mechanisms; inhibition of cell wall synthesis (β-lactams), inhibition of protein synthesis (aminoglycosides, tetracyclines), inhibition of nucleic acid synthesis (quinolones) or inhibition of metabolic pathways (sulfonamides). However, the main problem with this therapeutic approach is the development of new acquired resistances. These usually involve mutations in genes of the antibiotic target or acquisition of foreign DNA, allowing antibiotic modification, destruction or efflux [1]. Therefore, the search for novel antibacterial-targets is of utmost importance.

Recent research has aimed at exploring potential targets within proteins involved in the regulation of the genetic expression. This includes proteins involved in bacterial adaptation to their host environment during infection, promoting their dissemination inside the organism. For instance expression of some virulence factors is switched on when the pathogen penetrates inside the host environment [2]. Such an adaptation allows the infection to progress and bacteria to proliferate. To achieve this goal, bacteria usually operate at two main levels of gene expression regulation: (i) regulation at the transcriptional level. Nucleoid-associated proteins for instance are involved in the maintenance of bacterial chromosomal architecture. They affect the expression of a large number of genes, most coding for housekeeping-proteins, but also for proteins involved in response to environmental changes induced during host colonisation [3,4,5,6]; (ii) regulation at the post-transcriptional level [7]. One of the most promising approaches in this direction consists in blocking noncoding RNA-based (ncRNA) regulation [8,9]. Many ncRNA from different bacterial species have been identified. They are on average 100 nucleotides long, hence their name of small RNA (sRNA) [10,11]. In Gram-negative bacteria, sRNA often functions by base pairing within regions around the translation initiation signal of the associated mRNA target, and therefore acts on both mRNA translation and stability [12]. In vivo, a protein called Hfq is required for this sRNA-regulation as it promotes annealing of the regulatory sRNA to its cognate mRNA [13,14]. Due to the diversity of their mRNA targets, sRNA and Hfq are thus involved in important bacterial cell processes, including virulence, quorum sensing and pathogenicity [15,16,17]. Taking into account the crucial role of this protein in the adaptation to the host environment, blocking Hfq function may depress Gram-negative bacterial adaptive capacity, including resistance to antibiotics [18,19]. Efforts have thus been made to block Hfq function in order to affect bacterial virulence [19].

Interestingly, Hfq is structurally related to the Sm eukaryotic family of proteins, which participates in RNA-related processes such as splicing, decapping and decay [20,21]. Indeed, the amino-terminal region of Hfq (NTR, about 65 amino acid residues) folds similarly to Sm proteins. This region comprises a bent antiparallel β-sheet, capped by an N-terminal α-helix. The β-sheets from six monomers interact with each other to assemble in a hexameric toroidal structure [22,23]. It is established that the inner pore on the proximal face of the NTR torus (on which the α-helix is present) binds U-rich RNA, while A-rich sequences bind to the distal face [24,25,26]. Attempts have already been made to block Hfq-NTR interaction with sRNA, in order to affect virulence [19]. Nevertheless, besides its Sm-like domain, the Hfq C-terminal region (CTR) also plays a role in nucleic acid recognition [27,28]. While numerous 3D-structures of various Hfq have been resolved [25,26,29,30,31,32], until now all lack the CTR, and the way this region folds remains unknown [23]. It has however been shown that Hfq CTR is able to self-assemble into an amyloid-like structure [33,34]. Furthermore, this amyloid-like region dictates Hfq cellular location, namely at the periphery of the bacterial cell, near the inner membrane [34,35,36]. Almost one half of Hfq-binding sRNAs with known targets regulate the expression of membrane proteins [37], allowing exchange with the extracellular environment. Therefore Hfq self-assembly, which targets Hfq to the membrane, may be of primary importance for its role in bacterial cell adaptation [38]. We thus hypothesize that interfering with the formation of the Hfq-CTR amyloid structure may have direct consequence for bacterial survival.

Epigallocatechin-3-gallate (EGCG) is the major polyphenolic catechin found in green tea (*Camellia sinensis*). EGCG has been shown to inhibit fibrillation of amyloidogenic proteins, such as Aβ or Sup35 [39]. Besides the known effects of EGCG on amyloidogenic proteins associated with diseases, EGCG also binds to functional amyloids that perform physiological roles [40]. In this work, we intend to analyse the effect of EGCG on bacterial Hfq functional amyloid, with the aim to impede bacterial adaptation. Blocking the formation of Hfq-CTR using small molecules may indeed be a promising strategy for antibacterial development that may be used synergistically with conventional antibiotics.

## 2. Results

### 2.1. EGCG Remodels Hfq CTR Amyloid Fibrils

**Figure 1:**
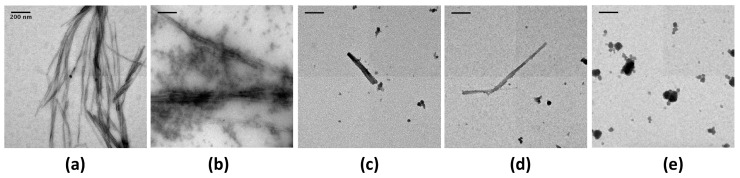
Transmission electron microscopy (TEM) visualisation of the effect of epigallocatechin-3-gallate (EGCG) on CTR_11_ preformed fibrils. (**a**) CTR_11_ without EGCG (control); (**b**) CTR_11_ with 1 mM EGCG; (**c**) CTR_11_ with 2.5 mM EGCG; (**d**) CTR_11_ with 5 mM EGCG; (**e**) EGCG 5 mM (no peptide). The presence of 1 mM EGCG is sufficient to dissociate the fibrils, even if the effect is not complete (incubation time 24 h). At 5 mM only a few fibrils remain on the grid (**d**). The images are representative of several tens of images that have been collected in each condition. Scale bars, 200 nm.

**Figure 2:**
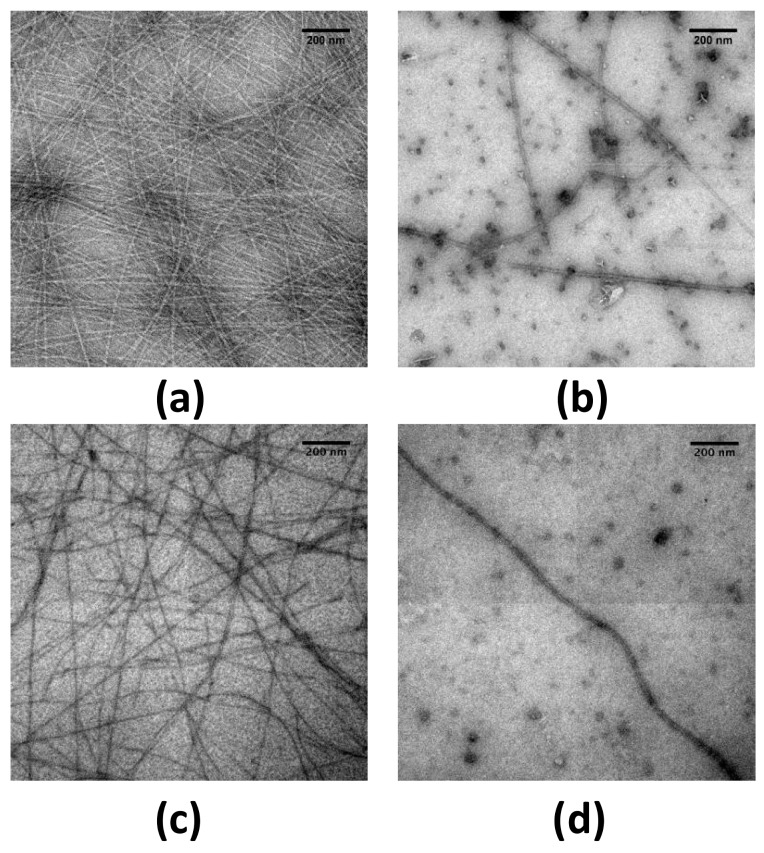
Transmission electron microscopy (TEM) visualisation of the effect of epigallocatechin-3-gallate (EGCG) on CTR_38_ fibrils. Upper panel (**a**,**b**) pre-assembled fibrils of CTR_38_ alone; lower panel (**c**,**d**): fibrils of CTR_38_ formed in the presence of DNA. (**a**) CTR_38_ (control); (**b**) CTR_38_ with 5 mM EGCG; (**c**) CTR_38_ + DNA (control); (**d**): CTR_38_ + DNA with 5 mM EGCG. Incubation time with EGCG was 24 h. Scale bars, 200 nm.

To investigate the possibility that Hfq functional amyloids can be remodeled in vitro, we screened the effect of EGCG on Hfq-CTR fibrils using transmission electron microscopy (TEM, see Section 4.3). In order to allow a statistical analysis on the sample, we used a negative staining procedure and not cryoTEM to reduce the length of acquisition. Note that thioflavin T staining is a widely used method to follow amyloids self-assembly. Nevertheless in the case of Hfq-CTR, thioflavin inhibits fibril formation and cannot be used [41]. As a model we first used the 11 amino acid residues peptide corresponding to the Hfq amyloid region only, referred to as Hfq CTR_11_ [42] (see sequence in Materials and Methods, Section 4.2). The effect of the EGCG’s potential amyloid interference was tested on preformed CTR_11_ fibrils (50 mg/mL). Fibrils were incubated in EGCG solutions (from 1 to 5 mM) for 24 h and examined by TEM (Figure 1). In the absence of the compound, the fibrils were abundant (Figure 1a), while incubation with 1 mM EGCG significantly reduced the amount of fibrils and led to formation of diffuse aggregates close to the fibrils (Figure 1b). At higher concentrations only rare fibrils can be observed (Figure 1c,d). This effect was confirmed with the 38 amino acid residues peptide corresponding to the full Hfq-CTR region (Figure 2). The corresponding peptide is referred to as Hfq CTR_38_ [34] (sequence in Materials and Methods, Section 4.2). TEM analyses did not allow for observing the total disappearance of CTR_38_ pre-formed fibrils when EGCG was added, in contrast to Hfq CTR_11_. Nevertheless, we clearly see lower amount of fibrils, which confirms that EGCG also disrupts CTR_38_ fibrils, even if less efficiently than in the case of CTR_11_ (Figure 2a,b) Note that spontaneous self-assembly of CTR_38_ is significantly lower than for CTR_11_ (weeks vs days) and that CTR_38_ peptide has been incubated for a few weeks to assure self-assembly before adding EGCG for 24 h [34,43].

To know whether EGCG can inhibit the formation of new fibrils and not only disrupt pre-formed ones, its effect on the conversion of Hfq monomers into amyloid fibrils was also investigated. This analysis was not possible with CTR_11_, as this peptide forms fibrils within a few hours and EGCG disrupts pre-formed fibrils in the same timeframe. We thus used Hfq CTR_38_ fibrils, which are more stable, for our analysis. To induce fibrillation of CTR_38_ within a shorter time (few days) we added DNA, which has been shown to accelerate fibril formation [43]. TEM analyses showed EGCG inhibition of the formation of Hfq CTR_38_ (Figure 2c,d). This indicates that EGCG does not only disrupt Hfq CTR preformed fibrils, but also inhibits their fibrillation.

### 2.2. Characterization of Hfq Amyloid Region Stability in the Presence of EGCG

**Figure 3:**
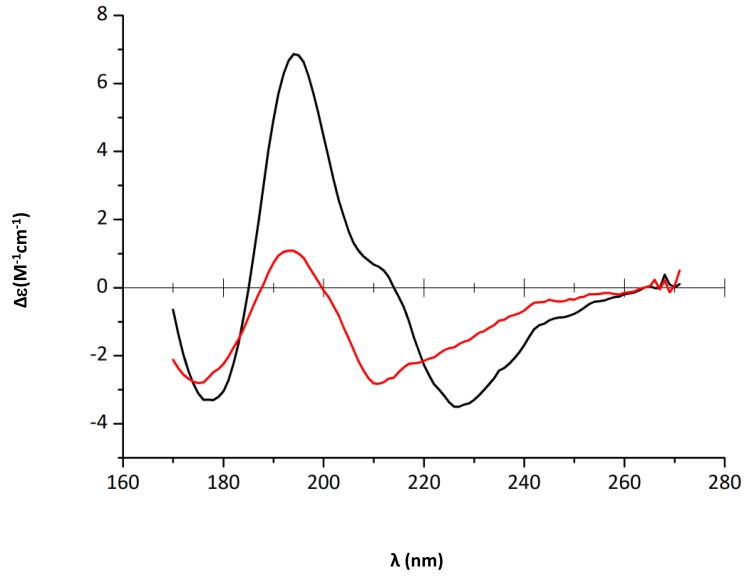
CTR_11_ disassembly in the presence of epigallocatechin-3-gallate (EGCG) 5 mM (black: 0 h and red: 4 h). Dissociation of the amyloid structure is observed by a shift of the negative peak from 226 to 212 nm [47]. The decrease of peak intensities also confirms disassembly. Path length = 1 µm, peptide concentration 12.5 mg/mL.

**Figure 4:**
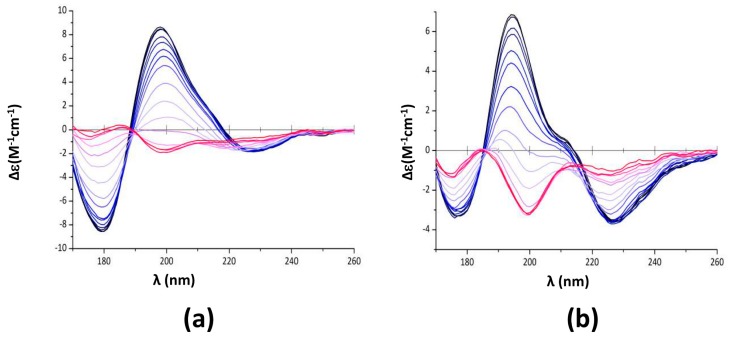

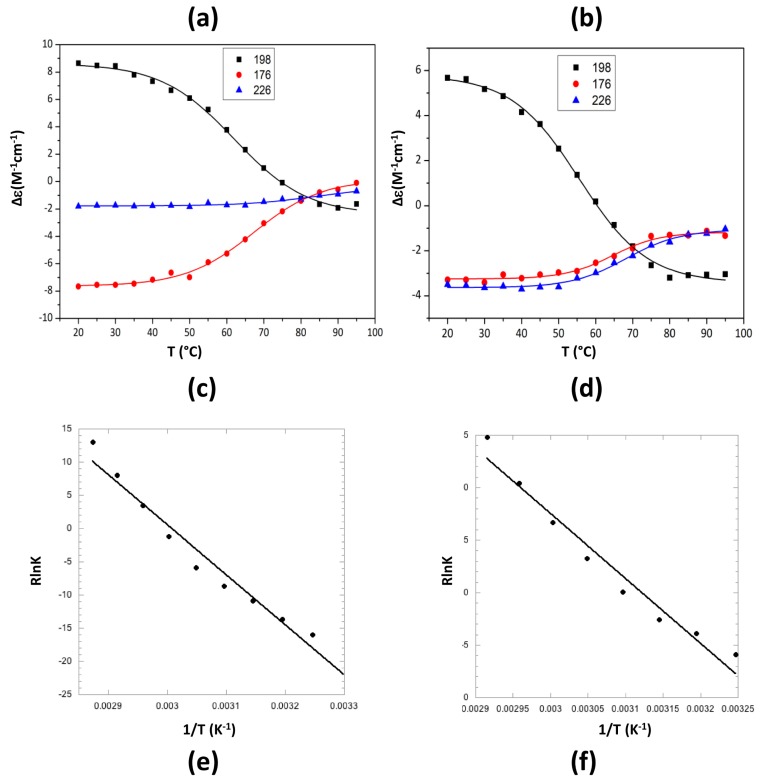
Melting curves of CTR_11_ fibrils, alone or in the presence of epigallocatechin-3-gallate (EGCG). (**a**,**c**,**e**) CTR_11_ alone; (**b**,**d**,**f**) CTR_11_ in the presence of EGCG 5 mM (substracted from EGCG signal). (**a**,**b**) Synchrotron radiation circular dichroism (SRCD) spectra measurements were carried out every 5 °C; blue 20 °C; red 95 °C (**c**,**d**): Measurement of the melting temperature at 176, 198 and 226 nm. The inversion of the band at 198 nm from a positive to a negative value is the signature of the conversion from relaxed antiparallel β-sheets (β anti 2) into right-handed twisted antiparallel β-sheets (β anti 3) [47] (Table 2). The melting temperatures measured from SRCD values at 198 nm are Tm = 62.0 ± 1 °C for CTR_11_ alone (**c**) and Tm = 55.6 ± 1 °C in the presence of EGCG (**d**). (**e**,**f**) Van’t Hoff’s plots giving access to ∆H° and ∆S° of CTR_11_ melting. The isosbestic point indicates a two state model and a Boltzmann sigmoid equation has been used for melting curves fitting.

We then analysed quantitatively the effect of EGCG on Hfq CTR stability. For this goal we used circular dichroism (CD). Precisely, we used synchrotron radiation circular dichroism (SRCD) for our analysis, which allows the wavelength range to be extended down to 170 nm for the identification and distinction of amyloid signals [44] (see Section 4.4). Aggregation into β-sheets in an amyloidal structure implies significant SRCD spectral changes: inversion of the band at ~200 nm from a positive to a negative value (Figure 3 and Figure 4); in parallel, a negative band decreases and shifts from ~225 nm to ~210 nm (Figure 3). The inversion of the band at ~200 nm and the blue-shift at 225 nm observed upon EGCG addition (5 mM, Figure 3) proves that the amyloid structure is disrupted by the compound, while the same peptide in the absence of the compound remains assembled. Precisely, in the presence of EGCG, relaxed antiparallel β-sheets (referred to as β anti 2, [45]) convert into right-handed twisted antiparallel β-sheets (referred to as β anti 3, [46]) (Table 1) [47].

Note that a similar effect is also observed with Hfq CTR_38_, even if in this case the blue shift is less noticeable (not shown). To quantify this effect on CTR_11_, we then analysed SRCD melting curves of preformed fibrils of CTR_11_ in the presence of EGCG (Figure 4).

The respective secondary structure compositions at different temperatures were determined using Bestsel and are reported in Table 2. As expected, at room temperature the peptide is partially folded into β-sheets, mainly in relaxed antiparallel β-sheets (β anti 2) [47]. Note that adding EGCG immediately results in the β anti 3 structure melting at 20 °C (Table 2).

When temperature increases, relaxed antiparallel β-sheets (β anti 2) convert into right-handed twisted antiparallel β-sheets (β anti 3) [47] (Table 2). The signature of this conversion is mainly observed at 198 nm (Figure 4a,b) [47]. We thus analysed the melting temperature of the assembly based on the SRCD signal at 198 nm. We confirmed the disrupting effect of epigallocatechin-3-gallate (EGCG) as melting temperature decreases significantly from Tm = 62 ± 1 to Tm = 55.6 ± 1 °C in the presence of EGCG (Figure 4b,d). The Tm in absence of EGCG was confirmed using differential scanning calorimetry (DSC) (Tm = 63.9 ± 1 °C, Supplementary Figure S1). DSC analysis in the presence of EGCG did not yield interpretable data as EGCG alone gives a DSC signal close to that of the peptide. Van’t Hoff’s plots were drawn from melting curves (Figure 4e,f), giving access to ∆H° and ∆S° of CTR_11_ fibrils disassembly in the absence or presence of EGCG. For CTR_11_ fibrils alone, ∆H° = 75.06 ± 4.5 kJ·mol^−1^ and ∆S° = 0.225 ± 0.13 kJ·mol^−1^K^−1^, while for CTR_11_ in the presence of EGCG ∆H° = 61.9 ± 4.9 kJ·mol^−1^ and ∆S° = 0.193 ± 0.015 kJ·mol^−1^K^−1^. In both cases, we confirm disassembly is an endothermic process. Furthermore, we confirm that fibrils are significantly less stable in the presence of EGCG.

### 2.3. EGCG Affects Bacterial Survival Due to Its Interaction with Hfq CTR

Finally, the effect of EGCG on bacterial survival has been evaluated. For this goal, different bacterial strains have been used, namely MG1655 WT cells [48], MG1655 ∆*hfq* and MG1655 *hfq72* (deleted for the last 90 hfq nucleotides giving a truncated protein with only the first 72 amino acids, this strain can also be referred to as *hfq∆ctr*, see Section 4.5) [43]. As EGCG is a highly absorbing and fluorescent molecule with spectral properties depending on solvents [49], the use of bacterial viability kits based on fluorescence or colorimetric assay was not possible for this analysis [50]. The plate count method has thus been used to evaluate the concentration of the antimicrobial agent, here EGCG, which inhibits bacterial growth. Precisely, colony forming units (CFU) have been measured and the C_50_, the concentration of EGCG that decreases CFU by 50%, has been determined (see Section 4.6). The C_50_ was 0.67 ± 0.02 mM for the WT strain vs 0.81 ± 0.05 mM for *hfq72*. Thus, the *E. coli* strain devoid of Hfq-CTR is less sensitive to EGCG than the WT strain. Note that due to the pleiotropic effect of the protein, the total absence of Hfq has a major effect on bacterial survival in the presence of EGCG (C_50_ ~0.4 ± 0.1 mM). This was expected as Hfq and sRNA establish resistance to various drugs, including antibiotics, in bacteria [9,18,51].

## 3. Discussion

Green tea polyphenolic catechins, including EGCG, have been shown to have antibacterial properties [52]. These properties arise from different mechanisms, including damage to bacterial cell membrane, inhibition of fatty acid synthesis, inhibition of enzyme activities (DNA gyrase, dihydrofolate reductase, etc) or act as an antibiofilm agent [53,54,55,56]. In this work we show that in addition to these roles, the EGCG catechin is also able to block the adaptability of bacteria when colonizing its host. Precisely, EGCG acts on the *E. coli* Hfq protein and inhibits its self-assembly [34]. Note that antibacterial effects reported previously for EGCG may be partially attributed to the inhibition of Hfq function, precisely in membrane and DNA-related processes or in biofilm formation [56,57,58].

In this work, we fully characterize the effect of EGCG on Hfq amyloid region self-assembly. As expected, we observe that Hfq-CTR amyloid disassembly is an endothermic process and we show that adding EGCG significantly decreases the enthalpy of the reaction (by ~20%). This suggests that EGCG acts on CTR_11_ oligomers mainly through disruption of hydrogen bonds. Hydrophobic interactions (entropy-driven) do not seem to be significantly involved. This is however not surprising taking into account the low number of hydrophobic amino acid residues present in the amyloidogenic region of Hfq [59]. Even if EGCG, the natural product, emerges as a promising new antibacterial agent against Gram-negative bacteria and exhibits alone antibacterial activity, this compound may be improved to more specifically and efficiently target the Hfq amyloid region. Catechins from other plants may alternatively be more specific. Otherwise, the galloyl moiety of EGCG could be chemically modified with the perspective of having more specific anti-Hfq properties [60]. Note that the in vitro inhibition concentration and C_50_ values we measured (in the mM range) are close to the minimal inhibitory concentration (MIC) reported for other Gram-negative bacteria (about 1 mM, [61,62]). MICs in Gram-negative bacteria are significantly higher than that in Gram-positive bacteria, probably due to protection by the outer membrane [62]. This C_50_ concentration is significantly higher than the equilibrium dissociation constant (K_D_) of the EGCG/CTR_11_ complex, which is in the µM range (Supplementary Figure S2). This suggests that the property of EGCG that must be optimised is its disassembling efficiency, and not its binding to CTR_11_. Nevertheless, the precise 3D structure of this region of the protein is still unknown [23], and rational design of modified catechins to target Hfq-CTR is thus challenging.

## 4. Materials and Methods

### 4.1. Chemicals

All chemicals including EGCG were from Sigma-Aldrich. EGCG was prepared in milliQ water at 10 mM.

### 4.2. Hfq CTR Peptides

Hfq CTR_38_ and CTR_11_ peptide were chemically synthetized (Proteogenix SA) [34]. Corresponding sequences were for CTR_38_: SRPVSHHSNNAGGGTSSNYHHGSSAQNTSAQQDSEETE, and for CTR_11_: SAQNTSAQQDS. The fibrils were obtained from a solution at 20 or 100 mg/mL incubated for days (CTR_11_) to weeks (CTR_38_). DNA-induced fibrillation was obtained in the presence of (dA:dT)_59_ duplex (2 mM) (Eurogentec), as described in Malabirade et al. [43].

### 4.3. TEM Imaging of Protein Fibrils

EGCG was added to fibrils (final concentration from 1 to 5 mM). Except when specified, samples were incubated for 24 h and visualized by TEM. To perform negative staining, 5 μL of peptide sample was deposited on a glow-discharged carbon-coated electron microscopy copper grid (continuous carbon film on 200 mesh square copper grid, EMS). After 2 min, the excess of sample was blotted out using a Whatman filter paper. Then, 5 μL of contrasting agent solution (Gadolinium salt, uranyl-less) was applied onto the grid with peptide. After 1 min incubation, the excess of contrasting agent was blotted out and then the grids were kept in a dry dark dust-free environment until observation with the electron microscope. For sample observation, the electron microscopy grid was mounted onto a room temperature holder and introduced into a JEOL 2200FS electron microscope (JEOL, Tokyo, Japan). The TEM images (2048 × 2048 pixels) were acquired using a Gatan UltraScan894 US1000 slow scan CCD camera at 40,000× nominal magnification (corresponding pixel size was 0.32 nm). The images presented in this work are representative of several tens of images that have been collected on 2 independent samples.

### 4.4. Synchrotron Radiation Circular Dichroism (SRCD)

For SRCD analysis, measurements and data collection were carried out on DISCO beam-line at the SOLEIL Synchrotron (proposal # 20180165) [63]. Samples of 2–4 µL were loaded into circular demountable CaF_2_ cells of 1 micron path length and analysed at different incubation time [64]. Separated data collections were carried out to ensure repeatability. Spectral acquisitions of 1 nm steps at 1.2 integration time, between 320 and 180 nm were performed in triplicate for the samples as well as for the baselines. (+)-camphor-10-sulfonic acid (CSA) was used to calibrate amplitudes and wavelength positions of the SRCD experiment. Data-analyses including averaging, baseline subtraction, smoothing, scaling and standardization were carried out with CDtool. Secondary structure content was determined using BestSel [47,65]. Normalized root-mean-square deviation (NRMSD) indicated the most accurate fit for each spectrum; values of <0.15 were considered significant. For melting curves, SRCD spectra were acquired every 5 °C between 20 °C and 95° C (in 1 micron path length cell) for CTR_11_, CTR_11_ in the presence of EGCG and EGCG alone. SRCD values at 198 nm are presented as a function of temperature to measure the melting point (Tm). A Boltzmann sigmoid equation, which assumes a two state model, has been used for fitting of melting curves: y = Bottom + (top − bottom)/(1 + e((Tm − x)/slope)). The energetics involved in self-assembly was determined by a van’t Hoff plot. Fractions of folded (f_n_) or unfolded (f_u_) structures were measured from melting curves. The equilibrium constant K was calculated as f_u_/f_n_. ΔG° the effective change in standard free energy of reaction is defined as ∆G° = ∆H° –T∆S° = -RT ln K, where ΔH° is the change in standard enthalpy of reaction, ΔS° the change in standard entropy of reaction, R the gaz constant and T the absolute temperature (expressed in Kelvin). ∆S° and ∆H° are extracted directly from the van’t Hoff plot where R lnK is plotted against 1/T (R lnK = ∆S° − ∆H° × 1/T), with −∆H° the slope and ∆S° the intercept at origin of the straight-line. Note that thermodynamic parameters may have an incertitude of ~10% due to the uncertainty for the lower and upper limit of the melting curves.

### 4.5. Construction of E. coli Strains

Strains were constructed with the λ-red recombination technique, as described in Malabirade et al. [43].

### 4.6. Effect of EGCG on E. coli Survival

The plate count method has been used to evaluate the efficiency of EGCG on bacterial EGCG. This method involves the incorporation of different concentrations of the antimicrobial substance into a LB agar medium followed by the application of a standardized number of cells to the surface of the agar plate [66]. Briefly in our case *E. coli* MG1655 strains were grown overnight in LB medium (37 °C at 120 rpm), diluted, and grown to an OD_600_ of 0.6. Diluted samples (from 10^−5^ to 10^−7^) were plated on agar plates in triplicate after overnight incubation. The experiment was repeated twice with independent cultures. Colonies were counted to determine CFU, an estimate of viable bacteria. To ensure no colonies grow slowly, plates were kept at 37 °C for 2 extra days. The concentration of the assayed antimicrobial agent (here EGCG) that decreases CFU by 50% has been measured in each case to evaluate the activity of EGCG as an antimicrobial agent. This value is referred as C_50_ in this manuscript.

## 5. Conclusions

Natural or modified EGCG are already known to work synergistically with other antibiotics against *E. coli*, *K. pneumoniae* or *A. baumanii* isolates. Here we show that they may be used efficiently with conventional antibiotics (such as β-lactams or chloramphenicol) to block bacterial adaptation [67,68,69,70]. This should allow extending the spectrum of agents against multi-resistant and biofilm forming bacteria, and in general provide an efficient anti-infective compound, especially as EGCG has also antifungal and antiviral properties [71].

## Figures and Tables

**Table 1:** CTR_11_ secondary structure contents in the presence of epigallocatechin-3-gallate (EGCG) at 0 and 4 h determined using BestSel [47].

Time (h)	Helix 1	Helix 2	Anti 1	Anti 2	Anti 3	Para	Turn	Others	NRMSD
CTR_11_ with EGCG 5 mM									
0	0.0	0.60	10.6	13.6	0.00	10.8	17.4	46.9	0.04649
4	5.1	5.8	0	4.8	11.4	12.1	15.4	45.3	0.06050

**Table 2:** CTR_11_ secondary structure content at various temperatures determined using BestSel [47].

Temp. °C	Helix 1	Helix 2	Anti 1	Anti 2	Anti 3	Para	Turn	Others	NRMSD
CTR_11_ without EGCG									
20	0.00	0.00	15.37	35.87	0.00	0.00	5.18	43.58	0.0403
25	0.00	0.00	15.31	35.95	0.00	0.00	5.38	43.36	0.0396
30	0.00	0.00	15.19	35.71	0.00	0.00	5.1	44.00	0.0404
35	0.00	0.00	14.87	33.75	0.00	0.00	5.46	45.92	0.0416
40	0.00	0.00	14.47	33.17	0.00	0.00	5.90	46.46	0.0406
45	0.00	0.00	14.01	31.45	0.00	0.00	6.35	48.19	0.0383
50	0.00	0.00	13.96	29.01	0.00	0.00	5.84	51.20	0.0434
55	0.00	0.00	12.15	27.17	1.15	0.00	7.57	51.96	0.0337
60	0.00	0.00	10.57	19.23	3.92	4.07	8.59	53.62	0.0360
65	0.00	0.00	8.73	13.38	7.00	9.34	10.06	51.49	0.0348
70	0.00	0.89	7.23	10.79	9.95	10.91	11.82	48.41	0.0352
75	0.00	1.85	5.77	8.62	11.43	11.72	13.07	47.53	0.0594
80	0.00	3.38	3.51	5.24	12.78	11.54	15.95	47.61	0.1453
85	2.33	3.29	3.75	7.39	13.70	7.38	15.53	46.62	0.0795
90	3.42	3.00	3.52	7.11	13.90	6.27	16.26	46.52	0.0780
CTR_11_ with EGCG 5 mM									
20	0.00	0.65	10.62	13.64	0.00	10.79	17.35	46.95	0.0467
25	0.00	0.85	10.27	13.10	0.00	11.97	17.12	46.69	0.0465
30	0.00	0.80	10.03	12.41	0.00	13.67	16.53	46.57	0.0496
35	0.14	0.77	10.08	12.61	0.06	11.63	17.07	47.63	0.0459
40	0.00	0.84	9.53	9.72	0.68	15.34	16.69	47.20	0.0595
45	0.95	0.70	9.52	9.69	2.01	12.69	16.66	47.79	0.0504
50	1.97	0.71	8.72	8.88	3.36	12.59	15.94	47.82	0.0554
55	3.22	0.56	7.85	8.86	5.68	10.53	15.60	47.69	0.0600
60	2.55	1.19	6.93	7.65	7.59	11.92	15.59	46.58	0.0750
65	2.41	1.13	6.19	7.57	9.72	10.31	15.75	46.91	0.0827
70	3.76	1.60	5.18	7.43	10.90	9.85	15.22	46.08	0.0921
75	2.74	1.91	4.30	7.84	12.47	6.95	16.54	47.26	0.0777
80	1.10	1.75	4.03	8.24	13.42	6.41	16.94	48.12	0.0629
85	0.00	1.69	3.91	9.64	14.08	4.95	16.79	48.94	0.0620
90	0.00	1.82	3.94	9.80	14.67	4.74	16.80	48.23	0.0655

## Supplementary Materials

The following are available online at http://www.mdpi.com/2076-0817/7/4/95/s1, Figure S1: DSC analysis of Hfq-CTR_11_ melting. Figure S2: Hfq-CTR11/EGCG equilibrium dissociation constant measurement using ITC.
