# Supplementary figures and images for: Epigallocatechin Gallate Remodelling of Hfq Amyloid-Like Region Affects Escherichia coli Survival
Source: Pathogens. 2018 Dec 1;7(4):95. doi: 10.3390/pathogens7040095 (PMC6313410; doi:10.3390/pathogens7040095)

ITC of CTER11 with ECGC

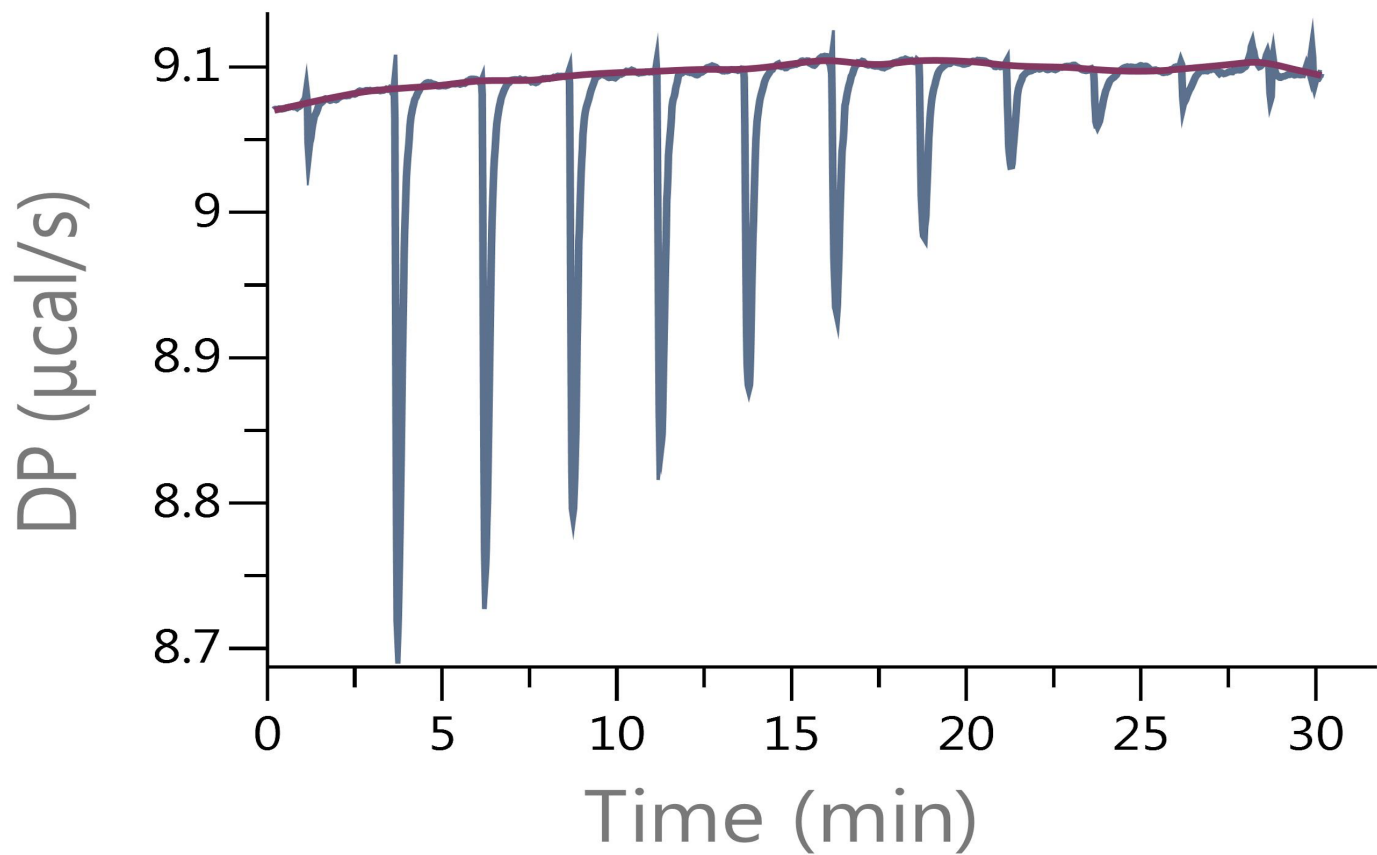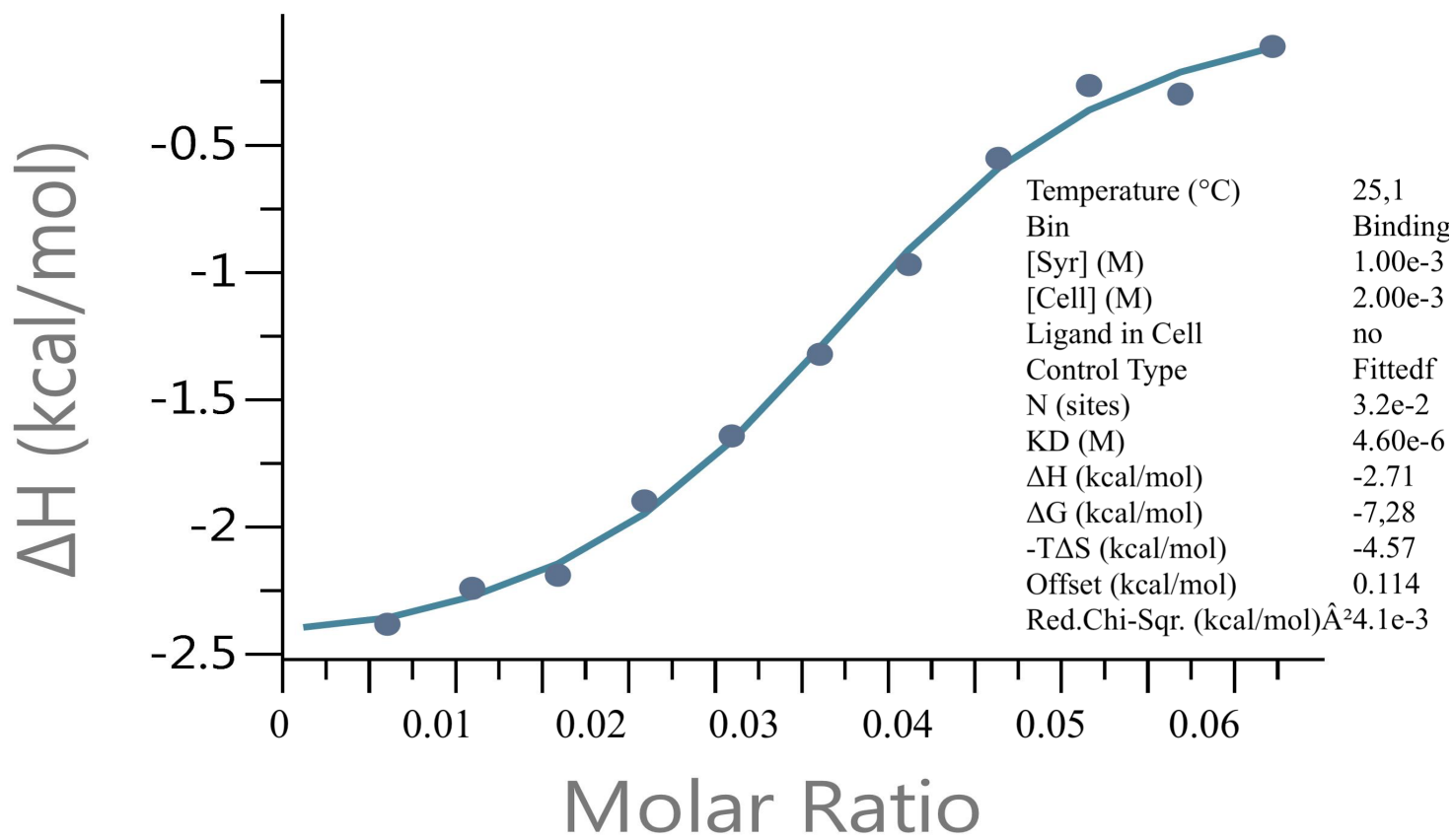

Supplement: Supplementary file 1 [file pathogens-07-00095-s001.zip › ITC.pdf]

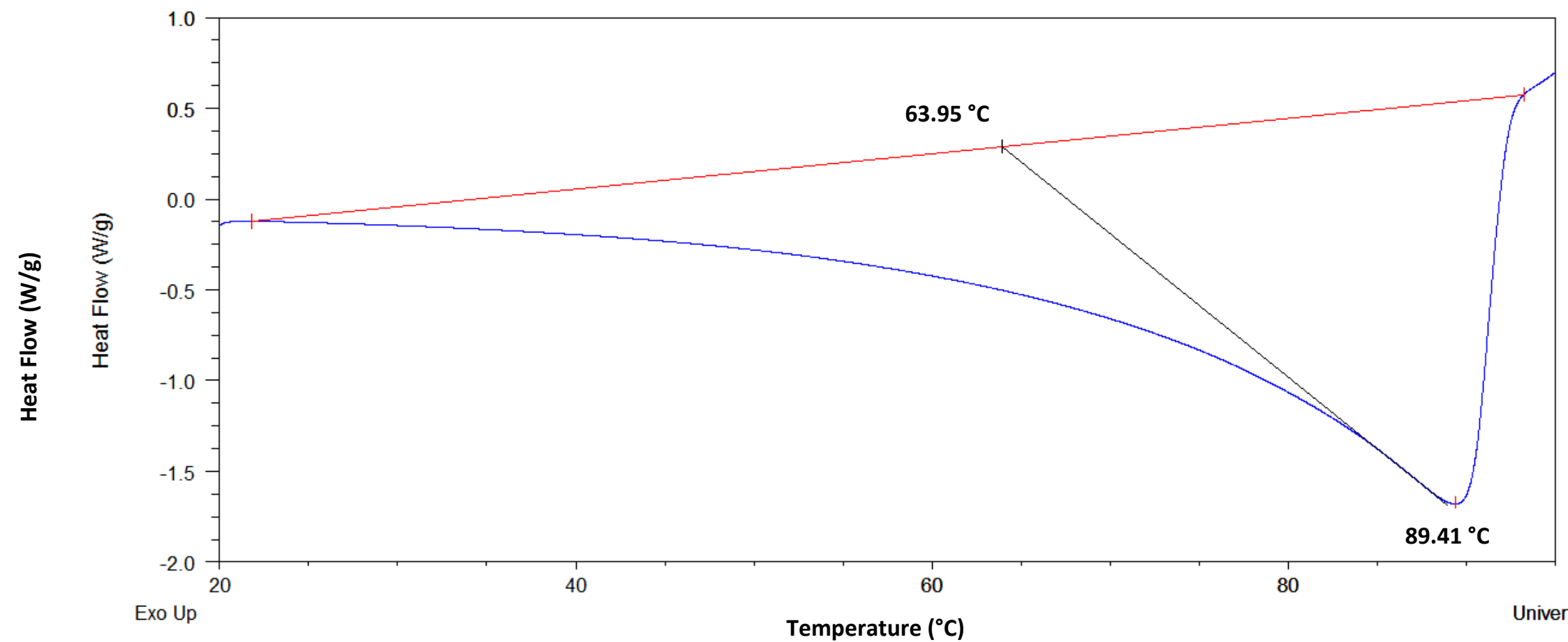

Supplement: Supplementary file 1 [file pathogens-07-00095-s001.zip › DSC.pdf]
